# 3D Multi-Modality Medical Imaging: Combining Anatomical and Infrared Thermal Images for 3D Reconstruction

**DOI:** 10.3390/s23031610

**Published:** 2023-02-01

**Authors:** Mauren Abreu de Souza, Daoana Carolaine Alka Cordeiro, Jonathan de Oliveira, Mateus Ferro Antunes de Oliveira, Beatriz Leandro Bonafini

**Affiliations:** Programa de Pós-Graduação em Tecnologia em Saúde (PPGTS), Pontifícia Universidade Católica do Paraná, Curitiba 80215-901, Brazil

**Keywords:** 3D thermo-scan, 3D models, 3D reconstruction, 3D visualization, infrared images, anatomy images, thyroid

## Abstract

Medical thermography provides an overview of the human body with two-dimensional (2D) information that assists the identification of temperature changes, based on the analysis of surface distribution. However, this approach lacks spatial depth information, which can be enhanced by adding multiple images or three-dimensional (3D) systems. Therefore, the methodology applied for this paper generates a 3D point cloud (from thermal infrared images), a 3D geometry model (from CT images), and the segmented inner anatomical structures. Thus, the following computational processing was employed: Structure from Motion (SfM), image registration, and alignment (affine transformation) between the 3D models obtained to combine and unify them. This paper presents the 3D reconstruction and visualization of the respective geometry of the neck/bust and inner anatomical structures (thyroid, trachea, veins, and arteries). Additionally, it shows the whole 3D thermal geometry in different anatomical sections (i.e., coronal, sagittal, and axial), allowing it to be further examined by a medical team, improving pathological assessments. The generation of 3D thermal anatomy models allows for a combined visualization, i.e., functional and anatomical images of the neck region, achieving encouraging results. These 3D models bring correlation of the inner and outer regions, which could improve biomedical applications and future diagnosis with such a methodology.

## 1. Introduction

Infrared thermography (IRT) presents different applications, ranging from engineering to biomedical applications [1,2,3,4]. In the medical field, assessing the amount of infrared radiation emitted by the human body can indicate normal or abnormal temperature distribution patterns. The use of medical thermography has the advantage of being contactless, enabling the visualization of temperature variations using thermal infrared cameras. In this scenario, it is possible to identify regions with high vascular activity, infection, tumor tissues, and muscle tensions. For example, infrared analysis identifies thermal variations, such as heat, redness, burning, local swelling, and high or low blood circulation in the examined body regions [4].

Computer systems and algorithms for medical diagnoses are becoming frequent since they favor further analysis and more accurate diagnoses and are less susceptible to errors. Doi [5] defines diagnostic support systems, such as CAD (Computer Aided Diagnosis), as a set of methodologies that predict the findings of lesions and abnormalities in images, describing patterns capable of estimating better diagnosis. 

Most medical thermography research comes from two-dimensional (2D) acquisitions, once only the standard temperature distribution is obtained from the thermal images and no spatial depth information is detected with single 2D infrared imaging modality. On the other hand, the depth information is perceived due to the photogrammetry approach from multiple images obtained at different positions/angles, which enables the calculation of a 3D point cloud. This is based on the stereo-vision from the camera positioning changes due to the parallax in space, which leads to 3D spatial information [6]. Then, computer vision techniques enable such procedures, covering imaging acquisition, processing, and analysis [7]. In addition to visible cameras, there are also the three-dimensional (3D) scanning or photogrammetric systems (either commercial or customized), which can generate 3D models of objects and body parts, adding crucial geometric details for image analysis and recognition, especially for biomedical applications.

The literature brings several references about the generation of 3D thermal imaging models, mainly focused on 3D thermography (i.e., only presenting the outer 3D thermal shell of interest and not including inner information) for different applications, which are worth mentioning. For example, Cabrelles et al. [8] showed the reconstruction of an archaeological monument in Petra (Jordan), generating 3D models (using both visible and thermal cameras). Yang et al. [9] presented customized equipment containing two smartphones and a low-cost thermal camera to acquire visual and thermal images. They also used the Structure from Motion (SfM) methodology, and the results presented were focused on the 3D thermal model reconstruction of buildings, facades, and concrete samples. Regarding another application, in the textile industry, Domina et al. [10] employed both 3D scanning systems and thermal images for mapping the 3D body scan of subjects together with their thermal variability, which focused on finding the woman’s thermal patterns. More recently, Campione et al. [11] presented a 3D thermal imaging system for cultural heritage applications, merging data from a generic thermal camera and a laser scanner.

Now focusing on biomedical applications, several approaches have used 3D thermography for diagnosing diabetic foot, such as Aksenov et al. [12], Ju [13], Barone et al. [14], and Parker et al. [15], among others. For example, Barone et al. [14] presented the integration of a customized 3D scanner (for geometrical data acquisition) together with a thermal image collection. Their focus is mainly on evaluating ulceration for diabetic foot disease. Parker et al. [15] also presented their thermal stereoscope system, employing three digital SLR cameras, an infrared camera, and a structured light pattern to be projected. The application was for foot surface reconstruction as well. 

Since there are several possible configurations for obtaining 3D thermograms, such as how many and which cameras (visible and thermal) are to be used, or the inclusion of 3D scanning systems (either custom-made or commercial systems), Chernov et al. [16] presented an overview of some of these systems. Additionally, they introduced their system, which comprises two units consisting of an off-the-shelf depth camera, rigidly mounted onto a FLIR thermal camera for biomedical applications. Cao et al. [17] introduced a robust mobile and real-time 3D thermographic reconstruction method through depth and thermal sensor fusion. In this configuration, their device consisted of a thermal camera and an RGB-D sensor, which enables the generation of 3D thermal models. They applied a customized method, called thermal-guided iterative closest point (T-ICP), which was compared to other established ICP algorithms. From our research group, Krefer et al. [18] presented a Structure from Motion (SfM) methodology, in which a decoupled system was employed, since it used 3D geometry (from a 3D commercial scanner as input) and the infrared images, acquired at different moments. Then, for proving the concept of the method, it was applied to a test object and some volunteers as well.

More recently, Bader [19] presented image processing and 3D reconstruction using the SfM method as well. His system consisted of two cameras in the visible spectral range and two thermal cameras. Doremalen et al. [20] presented a system composed of a high-resolution medical 3D imaging system—a 3D scanner (Vectra XT), aligned with three smartphone-based thermal infrared cameras. The purpose was the generation of 3D thermographs for inflammation detection in diabetic foot disease in a hospital environment. Mancilla et al. [21] employed a solution based on the SfM and Multi-view Stereo approach, to generate 3D thermal models, also focused on diagnosing diabetic foot. Schramm et al. [22] presented 3D thermograms and some advantages compared to other existing systems. Schmoll et al. [23] also presented 3D thermography describing the fusion of geometry and temperature sensors, but for heat dissipation applications.

Regarding thermal simulations, we provide just a brief presentation. For instance, Ledwon et al. [24] proposed a novel concept of the Convolutional Neural Network (CNN) on thermal tomography, which employed a reconstruction of the heat distribution from planar thermal images and synthetic design, for the validation of a model on a setup with a single heated object. Another recent study by Paz et al. [25] also explored a thermal reconstruction on a 3D model based on the segmentation of magnetic resonance images (MRI), to prove the concept of inner temperature propagations from a specific patient case, which was applied to detect changes in thyroid metabolism through the finite element analysis using ANSYS software. However, such thermal simulations and thermal tomography approaches are not the focus of this proposed research.

Additionally, based on the literature analysis, a variety of customized systems are observed, ranging from decoupled to combined systems. The integrated systems, which have infrared and visible cameras, provide a much more straightforward data combination, as both sensing devices are triggered together. On the other hand, the decoupled system approach allows the reuse of previously acquired data obtained at different moments or by additional modalities, for example, the anatomy medical images (such as MRI, CT, etc.). However, this is possible since it kept adequate data acquisition to maintain similar acquisition conditions (primarily related to spatial positioning and configuration of the body region to be examined), which is also a limitation for biomedical applications.

The aforementioned references are not exhaustive, since the focus here is not a deep review, but to demonstrate awareness of this topic. So, based on the applications from this brief literature review, it is concluded that there is still space for further development. Additionally, none of these previous papers had presented the inclusion of anatomy images. Therefore, the approach presented in this paper consists of medical applications, including the usual anatomy images (which are clinically already approved and employed in routine use) by the medical team.

On the topic of the 3D reconstruction of medical/anatomical images in DICOM format files, there are commercial software that perform such processing. So, the standard medical DICOM files acquired through computerized tomography (CT) or magnetic resonance imaging (MRI) are reconstructed, based on segmentation tools of the body structures under analysis. Examples are given, such as MIMICS^®^ and 3D Slicer^®^ software [26,27]. Using such an approach enables the 3D object model to be realistic and compatible with the real organ, resulting in the accurate volume and the perfect geometry. For example, Shin et al. [27] presented the importance of the 3D segmentation and reconstruction process, as it employed MIMICS^®^. So, it was shown how effective the segmentation and 3D reconstruction performed were. Additionally, Kaur & Jindal [28] used the same methodology for processing medical images.

Still, regarding the 3D reconstruction of objects/bodies by processing a set of 2D anatomy images, especially when merging and combining different medical imaging modalities (such as CT, MRI, and IRT), it is worth mentioning some studies. For instance, Souza et al. [29,30] first presented the original methodology of the 3D THERMO-SCAN, by using the SfM technique with a sequence of infrared images. Additionally, it a case study was presented for dentistry applications [30]. In these references, three imaging modalities were included: 2D infrared images, 3D scanned model, and the DICOM imaging slices. Then, as a result, a 3D thermal outer shell model combined with internal anatomy slices was obtained. Additionally, Schollemann et al. [31] presented the reconstruction and visualization of 3D mouse models showing internal anatomical information (based on the DICOM CT images) together with the 3D thermal outer shell, forming a multimodal imaging modality as well. These previous studies employed customized visualization software, primarily implemented for such purposes.

Then, within this established background, it is worth mentioning that these previous references [18,29,30,31] differ in some characteristics from the proposed research brought into this current paper. For example, they did not include the visualization of the inner structures presented here within well-segmented and delimitated anatomy Additionally, the 3D visualization tool originally employed in [18,29,30,31] had been changed; as in this research, we employed MeVisLab^®^ software (version 3.6.1), which provides much more options and support for 3D visualizations, as well as being open-source.

Therefore, there is space for further developments in this research field, allowing for the processing and generation of 3D thermal models, incorporating infrared images attached to the 3D geometry (inner and outer shell), focused on presenting a medical case study (neck/thyroid region). The proposed methodology performs correlations based on the superficial thermal changes, indicating the high activity of an organ/body and the correspondent connection with geometry structures (such as the segmented inner and outer anatomy), which are all unified and visualized employing MeVisLab^®^ software. 

Thus, the purpose of this research is to provide a unified 3D thermal anatomical model, containing data from different medical imaging sources, presenting computational processing. Therefore, all imaging processing and registration allows the medical team to perform further correlations between the superficial temperature changes obtained through infrared images and their correspondent geometry of interest (anatomical structures). The contribution of this paper is to provide the generation of a unique 3D thermal model—which is called the 3D THERMO-SCAN methodology [29]. Such an approach is based on the imaging registration and alignment between different imaging modalities (infrared functional and CT anatomical images), combined with the segmented anatomy structures of interest incorporated into the model. 

This paper structure is organized as follows: Section 2 describes the background information, presenting the Structured from Motion (SfM) and the Affine coordinates transformation. Section 3 presents the methodology—covering the data imaging acquisition (both infrared and anatomy images), the anatomy thresholding, the 3D imaging reconstruction, the 3D thermal geometry, the 3D registration, and the final 3D THERMO-SCAN model. Section 4 presents the results and their analyses, including the 3D visualization of the models obtained within the segmentation results, the generation of the 3D thermal model, and the complete visualization showing the final 3D THERMO-SCAN model. Lastly, Section 5 and Section 6 present the discussion and conclusions, respectively.

## 2. Background Information

A brief description of the concepts employed in this research is appropriate. So, we are presenting the process behind the Structure from Motion (SfM) approach and the image registration, mainly focusing on the Affine Transformation (applied between different 3D models) obtained from other imaging systems.

### 2.1. Structured from Motion (SfM)

Structure from Motion (SfM) is the process of 3D reconstruction from a series of images taken from different viewpoints, which has been employed over the years in computer vision, 3D scanning, augmented reality, among others [32,33]. This method commonly starts with feature extraction, matching, and geometric verification, which serves as the foundation for a 3D reconstruction stage that incrementally registers new images, performs the triangulation of the same scene points, filters outliers, and refines its reconstruction via bundle adjustment (BA) [33]. 

Regarding the SfM processing, this research employed the VisualSFM GUI application Open Source, initially developed by Wu [34,35], which consists of a group of techniques to generate a 3D point cloud via a 2D image compilation, collected throughout the whole imaged object. The steps used are described below.

- Compute Missing Matches (CMM)—make the images to converge on a plane. This image convergence comes from a two-dimensional plane into a three-dimensional plane corresponding to a 3D object. Through this CMM process, VisualSFM^®^ software interprets embedded 2D images and transforms them into a 3D object. Identifying the common features within all the images provided enough features to distinguish and detect the same mutual points.
- Sparse Reconstruction—based on the estimation of the points (coordinates) positioning, which is automated and performed by applying the SIFT (Scale Invariant Feature Transform) method [36]. Next, the features are combined to create a pool of coordinates in space, characterizing a point cloud. However, after generating the cloud, VisualSFM^®^ can erroneously present the position of the cameras in space, causing failures in generating the result from the cloud, so the third step is necessary (BA).
- Bundle Adjustment (BA)—corresponds to the camera positioning optimization in photogrammetry [32]. The algorithm is responsible for refining the 3D points into a geometric scene (i.e., into a 3D coordinate system). Additionally, the data obtained from the stereo-image pairs are approximated to the absolute values of the coordinates, optimizing, and decreasing errors in the point cloud generation.
- Dense Reconstruction—enables a point cloud to be generated that is completely dense and united. Additionally, to refine the point could, there is also another function called *Find More Points*, which enables the inclusion of additional points (coordinates) to provide a much denser point cloud.

To evaluate the correlation between pairs of images, where the aim is to justify the generation of a 3D model using 2D images, the VisualSFM^®^ employs the SIFT (Scale Invariant Feature Transform) algorithm. This algorithm combines common points between images and validates the distance of these points using the metric of the smallest Euclidean distance. In addition, there is a filter through RANSAC (Random Sample Consensus) for the matches found that are not consistent [18].

### 2.2. Affine Transformation

Image registration is fundamental, since it allows the mapping and transformation of two different images into a common coordinate system, through which it is possible to align and visualize both 3D models altogether [37,38].

The transformation process is required when images are acquired through different sensors, resolutions, or positions. Each registration method is formed by a set of equations, which transform one image’s coordinates into the other image’s coordinates [39].

There are different coordinate transformations such as identity, rigid, affine, and non-rigid. The main difference between non-rigid and rigid is the nature of the transformation. Rigid registration aims to find the six degrees of freedom, as can be seen in Equation (1), which maps any point in the source image to the corresponding point in the destination image [40]. Additionally, Equation (2) demonstrates an extension of the affine transformation model, which has twelve degrees of freedom and allows shear and scaling as well.


(1) $$T\left(x,\;y,\;z\right)=(\begin{array}{c} x\prime \\ y\prime \\ z\prime \\ 1 \end{array})=(\begin{array}{cccc} \cos\beta \cos\gamma & \cos\alpha \sin\gamma +\sin\alpha \sin\beta \cos\gamma & \sin\alpha \sin\gamma -\cos\alpha \sin\beta \cos\gamma & t_{x}\\ -\cos\beta \sin\gamma & \cos\alpha \cos\gamma -\sin\alpha \sin\beta \sin\gamma & \sin\alpha \cos\gamma +\cos\alpha \sin\beta \sin\gamma & t_{y}\\ \sin\beta & -\sin\alpha \cos\beta & \cos\alpha \cos\beta & t_{z}\\ 0 & 0 & 0 & 1 \end{array})\left(\begin{array}{c} x\\ y\\ z\\ 1 \end{array}\right)$$

(2) $$T\left(x,\;y,\;z\right)=\left(\begin{array}{c} x\prime \\ y\prime \\ z\prime \\ 1 \end{array}\right)=\left(\begin{array}{cccc} a_{00} & a_{01} & a_{02} & a_{03}\\ a_{10} & a_{11} & a_{12} & a_{13}\\ a_{20} & a_{21} & a_{22} & a_{23}\\ 0 & 0 & 0 & 1 \end{array}\right)\left(\begin{array}{c} x\\ y\\ z\\ 1 \end{array}\right)$$

## 3. Material and Methods

**Figure 1:**
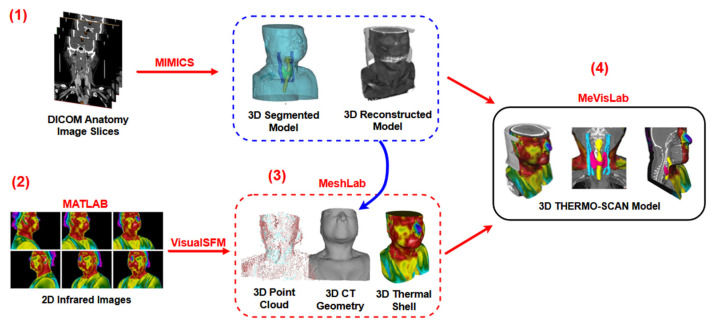
Schematic diagram of the proposed method.

The methodology applied in this research is summarized in the schematic diagram from Figure 1, contributing to an overall understanding of the proposed research. So, the central processing steps and their corresponding tools (software) are displayed: (1) From the DICOM anatomy images, a 3D model of the anatomic inner and outer structures is processed with MIMICS software. (2) From the 2D infrared images, a 3D point cloud is obtained using MATLAB^®^ and VisualSFM^®^ softwares. (3) Then, the image alignment between the 3D point cloud and the 3D model is performed, generating a 3D thermal model/shell (based on the corresponding thermal texture projection onto the 3D CT geometry) using MeshLab software. (4) Finally, it is possible to visualize all the 3D structures inside the 3D thermal geometry and to visualize the inner and outer models and the DICOM images altogether and simultaneously with MeVisLab^®^ software.

### 3.1. Data Imaging Acquisition

For the imaging acquisition, data is obtained from two different modalities: infrared (functional) images and computerized tomography (CT) (anatomy) images, which are detailed below.

#### 3.1.1. Infrared Images

For the infrared imaging acquisition, an acclimatization period of about 15 min is necessary. Then, the body region to be evaluated must be undressed. Thermal images were initially acquired via a FLIR thermal camera (model A325). Unlike most modern cameras, it is worth mentioning that this camera provides only infrared images and not visible images. The camera was positioned on a tripod, and the volunteers were placed in a swivel chair, where a complete video was collected going from the left to the right side (covering approximately 180°).

The acquisition of several thermal images of the neck region made it possible to evaluate and analyze these images for clinical assessment (obtained in video format, SEQ). The manipulation of these images allows the proper conversion to other formats that are more appropriate for the computational processing in the sequence.

The infrared data contains information about temperature. However, the range is only useful for representation purposes and clinical evaluation (initially defined in FLIR Tools and implemented in MATLAB).

After collecting the images, the FLIR Tools software is used for initial manipulations in the data file. The obtained video is saved in “SEQ” file format and converted to a “CSV” file (using FLIR Tools software). With this configuration, the temperature of each pixel of the thermal image is saved in a text file (CSV). Then, by using MATLAB, the “CSV” file is converted to the “MAT” format, to facilitate further processing in MATLAB as well (Section 3.3.1).

#### 3.1.2. Anatomy Images

**Figure 2:**
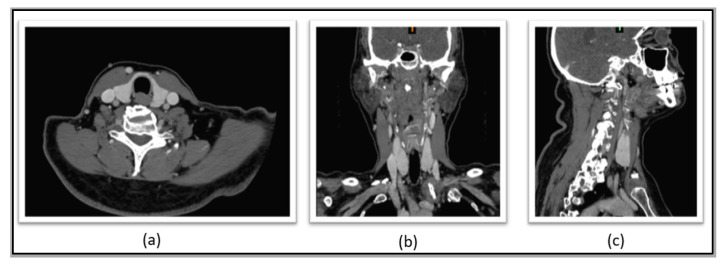
Thyroid seen in differentiated grayscale, which is shown in: (**a**) axial, (**b**) sagittal and (**c**) coronal.

Complementing the clinical protocol, patients underwent the acquisition of anatomical images (which can be either magnetic resonance imaging (MRI) or computerized tomography (CT)). To illustrate, Figure 2 shows some of the individual anatomical imaging planes (axial, coronal, and sagittal) obtained through the CT system. The CT equipment used in this research was a GE Optima CT660, with 64 channels, which allows acquisitions of 40 mm, providing isotropic images of 0.35 mm of spatial resolution. Additionally, for this CT acquisition, an iodine contrast is injected into the patient, so the thyroid gland is more evident in the images since the grayscale is better differentiated among the different anatomical structures. 

**Figure 3:**
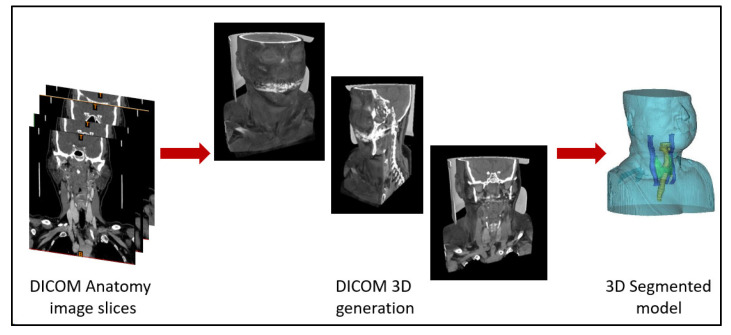
Representation of the DICOM anatomy imaging slices, illustrating the generation of the anatomy 3D generation and the correspondent segmented 3D model.

The purpose of the DICOM anatomical images is to provide inner information about the anatomy structures, which will be segmented (Section 3.2.1), to complement the generation of the 3D geometry model, as seen in Figure 3.

### 3.2. Anatomy Thresholding and 3D Imaging Reconstruction

#### 3.2.1. Segmentation of the Neck Region and Inner Structures

After the anatomical image data collection from CT (Section 3.1.2), a segmentation of the neck region (external geometry) and the inner structures was performed using the MIMICS Innovation Suite^®^ 17.0 software. The focus of this study is about the assessment of the thyroid gland, and for this reason, the following structures are segmented: thyroid, trachea, veins, arteries, and bust/neck.

**Figure 4:**
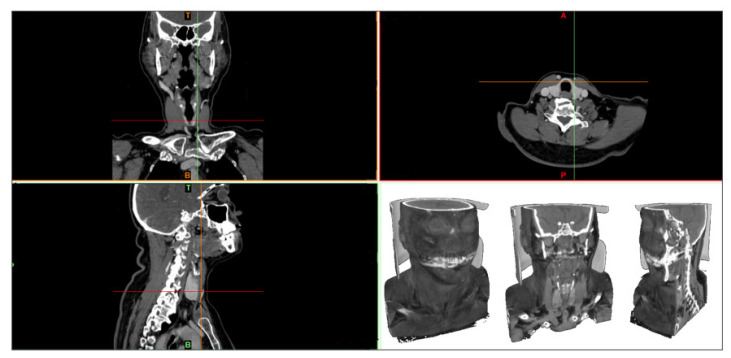
MIMICS software interface, where it is possible to observe the anatomical planes (sagittal, coronal, and axial).

The MIMICS software allows the visualization of the DICOM file, and the corresponding 3D reconstruction of these images [41]. The DICOM image set is a file that unifies and organizes the format of images obtained in such anatomy exams [42]. These images are imported into the software through the *New Project* option, which allows the visualization of each DICOM slice in the sagittal, coronal, and axial planes, where it is possible to navigate between them, as shown in Figure 4.

**Figure 5:**
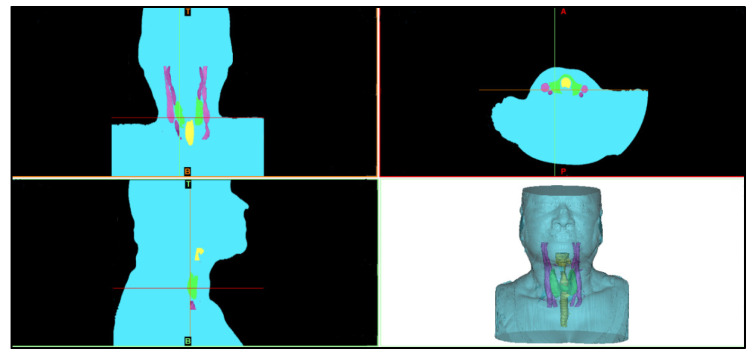
Visualization of the different masks (representing the different anatomic structures) and the complete segmented 3D model.

Through the axial imaging slices, a mask is created with the *Threshold* tool; thus, the edges of each structure in all the apparent layers are demarcated. After the delimitations, the software automatically fills in the gaps between the DICOM slices, thus forming a solid 3D object. Fault correction was performed using the *Multiple Slice Edit* tool, where removing or adding points of interest is possible. Figure 5 illustrates all the structures of interest with the mask and the complete 3D reconstruction.

After finishing the segmentation, the 3D model is exported to the Blender software (version 3.3), where the surface of the organs is smoothed, allowing their appearance to be as close as possible to the actual structures. 

It is noteworthy that the tools used in the Blender software are in the *Sculpting* mode, where the *Smooth* option performs the smoothing of the objects, and the *Fill* and *Clay* options fill in gaps present in the 3D object/model. All anatomy structures (thyroid, trachea, veins, arteries, and bust) undergo the same process.

### 3.3. 3D Thermal Geometry

#### 3.3.1. Infrared Imaging Pre-Processing

**Figure 6:**
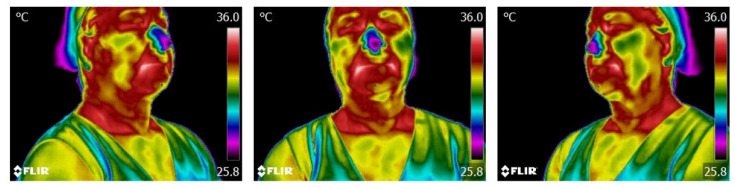
Thermal visualization representation in FLIR Tools^®^ at three different angles.

Afterward, the infrared images (frames) extracted from the thermal video undergo a pre-processing step (using FLIR Tools 6.4 and MATLAB). To illustrate, Figure 6 shows three samples (different frames) of the infrared data, represented in the Rainbow HC palette, which are initially visualized for clinical assessment, using the FLIR Tools^®^ software.

For the image treatment, some pre-processing steps were performed, as follows: (1) Import the CSV file (Comma Separated Values) previously generated in FLIR Tools^®^. (2) Generation of the frames (2D images). (3) Image intensification—a step characterized by a non-bijective transformation of the thermal representation in the image, which changes the color palette according to the previously recorded temperature. (4) Conversion of the files to a known image format (i.e., Portable Network Graphics—PNG).

The image intensification process (step (3)) consists of the same approach employed by Krefer et al. [18], in which a customized colormap is applied. Such transformation is based on changing the intensity levels of the thermal images (which are normalized in grayscale, and black and white (B/W) enhancements). This is performed to increase the number of regions with high-contrast texture and, consequently, the number of feature points detected. This customized intensification process is necessary since the thermal infrared images of the human skin do not have as many details as their corresponding visible images. Considering this and the fact that the SfM methodology was not initially designed for thermal images, such processing is essential for the proper performance of the SfM within such infrared images.

**Figure 7:**
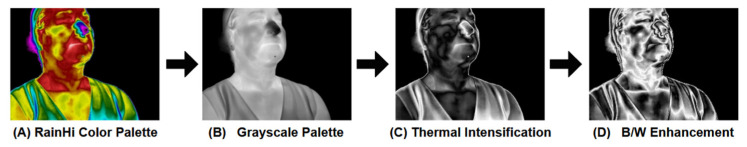
Representation of the image intensification process (step 3).

In this way, it is possible to improve the visual quality of the images to facilitate the generation of the 3D point cloud (SfM—Section 2.1). Figure 7 illustrates the resulting visualization of this intensity transformation. Thus, it summarizes the whole process, which is as follows: the most appropriate thermal palette is chosen, in this case, the RainHi color palette (represented by (A)—originally chosen using FLIR Tools). Next, the images are converted to a Grayscale Palette (B), which still represents low contrast in its palette. Then, Thermal Intensification (C) demonstrates an intermediate process, where such improvements are already perceived. The additional procedure in this intensification transformation presents stronger thermal differences, which is based on Black and White (B/W) enhancements (D) showing the whole image with much stronger contrast differences. These transformations are performed in MATLAB.

#### 3.3.2. SfM (Structured from Motion): 3D Point Cloud Generation

**Figure 8:**
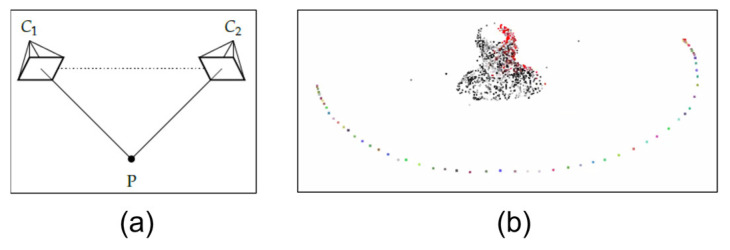
Illustration of the SfM technique: (**a**) representation of epipolar geometry; (**b**) result obtained from one of the mapped volunteers, showing the point cloud and the positioning of the cameras.

After pre-processing, the SfM (Structure from Motion) algorithm [43,44] is applied to generate a 3D point cloud from the thermal images. This step uses several images collected sequentially, allowing the selection of all frames. Applying the epipolar geometry shown in Figure 8a makes it possible to identify the same point in the two images [32]. Thus, the greater the correlation of the features between the images, the greater the number of corresponding points between each pair of images. Consequently, better 3D results are obtained, also denoted by Figure 8b.

In this research, the VisualSFM GUI application Open Source was employed allowing the 3D point cloud generation of the examined patient, with the 2D thermal images as input.

To generate the dense point could in the 3D space, obtained from the infrared images, processing using the VisualSFM^®^ application is employed, which is applied in the following order: (1) Compute Missing Matches (CMM); (2) Sparse Reconstruction—based on the estimation of the points (coordinates) positioning, following the SIFT (Scale Invariant Feature Transform) method; (3) Bundle Adjustment (BA); and (4) Dense Reconstruction—which were previously described in Section 2.1.

The 3D point cloud is obtained as part of the SfM process, performed using the VisualSFM^®^ software. This way, it is possible to map both the object being imaged (photographed) and the camera’s positioning [29,30]. In addition, we perform a camera calibration process to guarantee the metrics. Then, information on the camera’s intrinsic parameters is also included (such as focal length, main point, and radial/tangential distortions).

### 3.4. 3D Registration: Between 3D Thermal Model and Anatomy Images

Following the SFM methodology, merging the point cloud (obtained from the thermal images) with the 3D outer geometry (obtained from the reconstruction of anatomical images, in this case from CT images) is necessary. Then, a 3D registration occurs through a manual alignment (Affine Registration) between the point cloud and the 3D geometry, using the tools from the MeshLab^®^ software.

**Figure 9:**
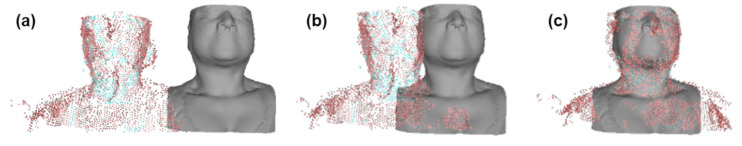
Alignment visualization: (**a**) pre-alignment; (**b**) during alignment; (**c**) post-alignment.

Thus, Figure 9 demonstrates the alignment process between the point cloud and the 3D geometry, which are finally presented in the same coordinate system and on the same scale.

**Figure 10:**
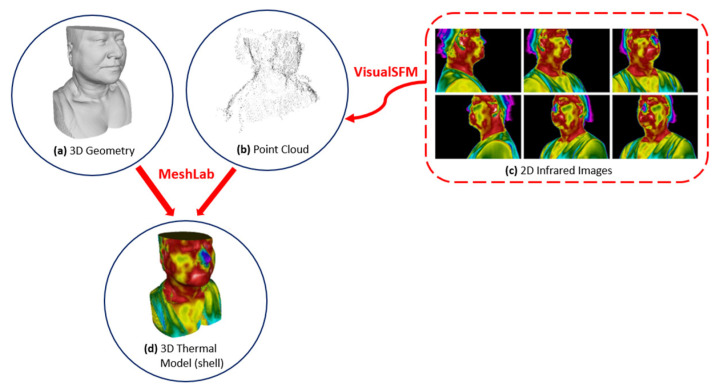
Flowchart showing the fusion and alignment of the imaging modalities: (**a**) 3D shell; (**b**) point cloud; and (**c**) thermal images, allowing the generation of the (**d**) 3D thermal shell.

The flowchart shown in Figure 10 illustrates the methodology used, in which it is possible to observe that the thermal images (c) represent the data input in the VisualSFM^®^ software. Therefore, the point cloud (b) is generated as part of detecting features obtained through thermal images (c). After this procedure, both the 3D shell (a) and the point cloud (b) are aligned, and the thermal images are projected onto the 3D outer surface, generating the 3D thermal shell (d).

**Figure 11:**
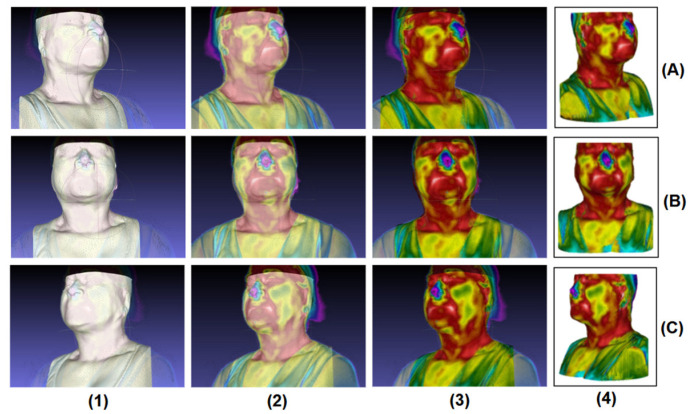
Texturing process steps: (1) 3D geometry (bust) segmented, without texture. (2) Applying the projection of the infrared images onto the 3D model (whole bust) showing a transparency. (3) Incorporation of the infrared images onto the 3D model. (4) Generation of the 3D thermal shell. These steps are represented by different positioning angles: (**A**) right side, (**B**) frontal and (**C**) left side of the volunteer.

Right after the alignment, it follows the thermal imaging projection (as represented in Figure 11), which is visualized within its inherent texture/thermal differences, generating what is called the 3D thermal shell/geometry. Detailing what arises from Figure 11, there is a demonstration of the texturing process on the previously segmented bust, in different imaging positions/views (frontal and lateral sides). Therefore, the steps of the texturing and projection process are: (1) the representation of the bust (3D model) without thermal texturing; (2) the projection of the infrared images onto the 3D model (showing transparency); (3) the visualization of the projection with all the infrared images directly on the model; (4) finally, the generation of the 3D thermal shell.

### 3.5. 3D THERMO-SCAN Complete Visualization

**Figure 12:**
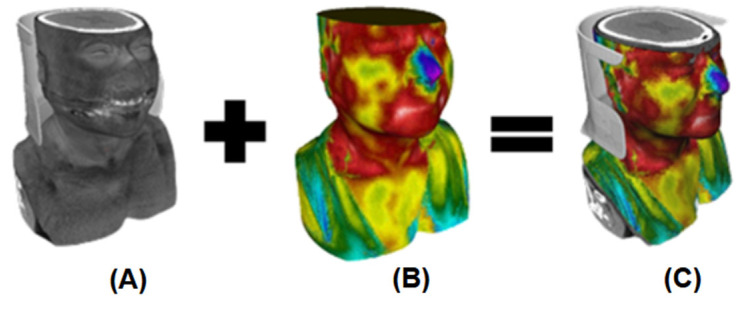
(**A**) 3D anatomy model generated from the 3D reconstruction (by the CT DICOM images). (**B**) 3D Thermal Model (obtained from the SfM methodology and its consequent thermal imaging projection onto the 3D outer bust model). (**C**) Combined registered 3D complete model—represented at the common coordinate system.

By using the interface of MeVisLab^®^ (version 3.6.1) [45], it is possible to generate a complete 3D model, which we called the 3D THERMO-SCAN model, due to the unification of the following models: (A) the 3D model obtained from the anatomic CT images (from Section 3.2—Figure 12A), combined with (B) the 3D thermal outer model (from Section 3.3—Figure 12B). This final 3D model allows the combined visualization altogether among the independent models, as shown in Section 3.5, leading to Figure 12C. Therefore, this research delivers the 3D combined visualization of several medical images into a common coordinate system. The new thermal and anatomical 3D model was saved into a 3D format (Polygon File Format (PLY) or Stanford Triangle Format (STL)). Then, it was imported into the MeVisLab^®^ interface for visualization purposes.

Therefore, the MeVisLab^®^ software can add more than one 3D object/model into the same coordinate system, even if they are in different file formats. However, to visualize the thermal shell with internal content (i.e., internal tissues and organs), it is necessary to include the DICOM image file that corresponds to the anatomical images. 

**Figure 13:**
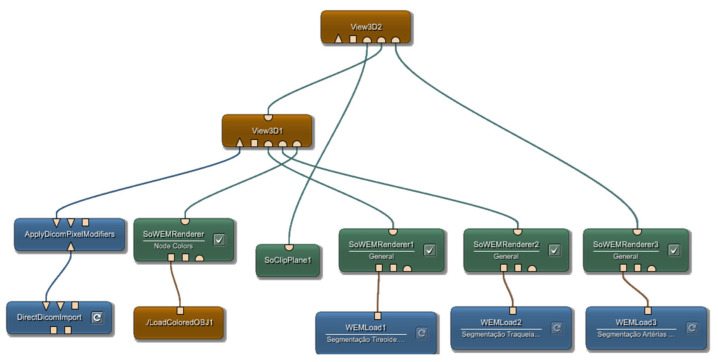
Representation of the network of modules used in MeVisLab^®^ to generate the complete 3D visualization.

The following data are included for the complete 3D model visualization: segmented 3D structures (thyroid, trachea, veins, and arteries), DICOM image slices, and the 3D outer thermal shell. These data are all joined into a unique coordinate system, which is merged/combined to allow complete 3D visualization. This fusion visualization takes place through programming modules presented in MeVisLab^®^ software, which performs the fusion of the various imaging modalities (3D thermal model geometry, the DICOM anatomy images, and the segmented inner structures). Figure 13 illustrates the programmed modules to allow the 3D visualization of the complete 3D model.

In these modules, there is a connection between them, and each one performs a distinct function. The blue modules in the lower left corner have the purpose of importing the DICOM files. Sequentially, looking from left to right, the following modules have the function of importing the 3D thermal shell (infrared) and rendering the object.

The rightmost blue and green pairs (*Load* and *Renderer*) have the functionality to import the previously designed segmentations, which, in this case, are the thyroid, trachea, veins, and arteries. Finally, the modules described above are coupled and put together in a visualization module (*View3D*). Thus, the final complete 3D model comprises the 3D thermal shell, anatomical images (DICOM), and the segmented inner structures (thyroid, trachea, veins and arteries).

## 4. Results

### 4.1. Segmentation Results

**Figure 14:**
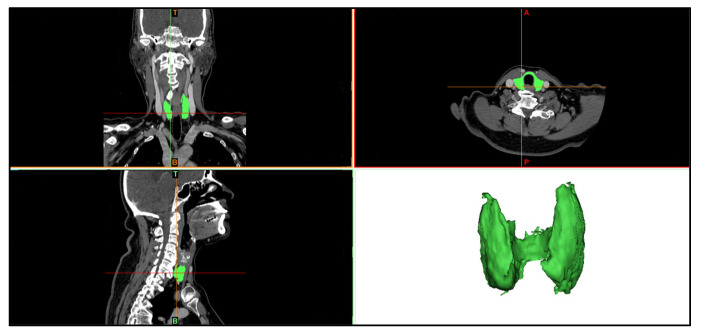
Illustration of a set of CT images visualized in the MIMICS software, to allow for the segmentation and 3D reconstruction of the thyroid geometry in the neck region.

Through CT images, it was possible to identify the thyroid gland and other structures in the anatomical sections (i.e., coronal, sagittal, and axial), as illustrated in Figure 14, evidencing the anatomical segmentation of the analyzed region. The 3D thyroid selection and reconstruction are highlighted in green in this image.

**Figure 15:**
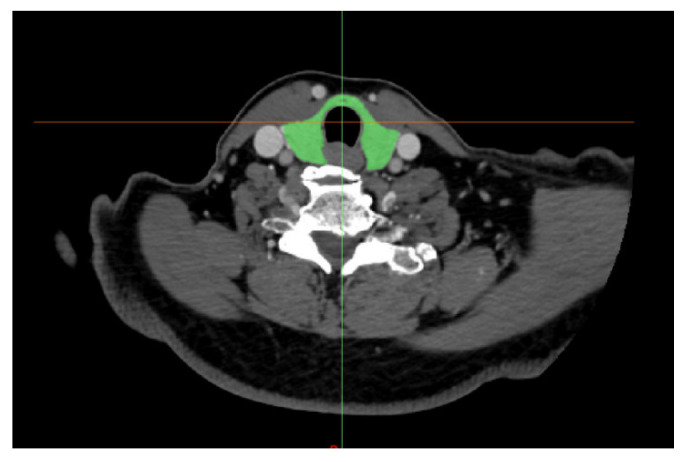
A highlight of an axial CT image, showing the delimitation of the segmentation mask (in green), applied over the structure to be segmented (i.e., in this case, the thyroid gland).

Figure 15 demonstrates one of the axial anatomical slices, illustrating the mask’s delimitation based on the thyroid gland’s thresholding in the correspondent slice. After including the mask in all the DICOM slices that show the thyroid, the software performed a filling between the slices, thus forming a 3D object with the entire gland’s geometry.

**Figure 16:**
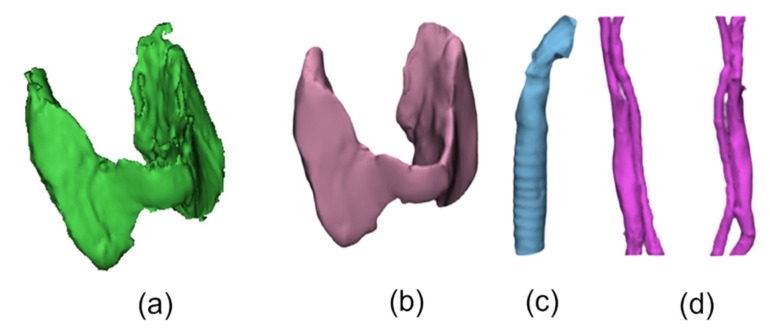
Representation of segmented inner anatomical structures: (**a**) thyroid (before smoothening/treatment) and (**b**) thyroid (after smoothening), (**c**) trachea, and (**d**) veins and arteries.

Usually, anatomical images contain noise, which can generate flaws in the reconstructed 3D model. Such noise can be attributed to various causes, such as the type of image (CT, MRI, etc.); additional accessories, such as orthodontic retainers, dental implants, and amalgams from dental restorations; movements of the patient/volunteer; among others. Due to these imperfections, it is necessary to improve and smooth the 3D model, which is transferred to the 3-Matic Medical software. Additionally, for a final 3D modeling treatment, the 3D model is also smoothed in the Blender software (version 3.3). The other anatomy structures were also segmented and smoothed with this process to correct possible failures. The results obtained from the thyroid, trachea, veins, and arteries are also shown in Figure 16. 

**Figure 17:**
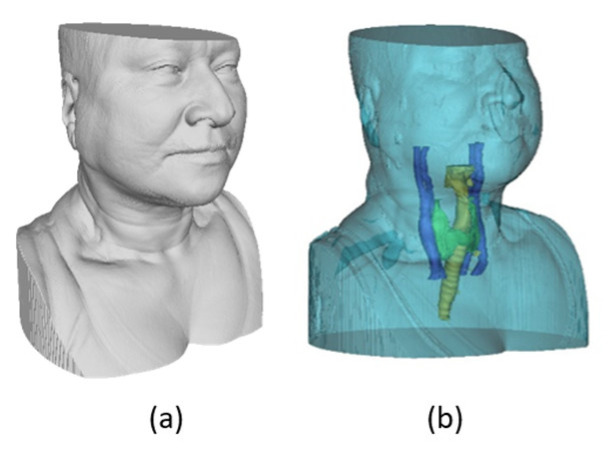
Segmentation results: (**a**) 3D geometry of the bust/neck (outer region only); (**b**) 3D model combined, showing all inner and outer anatomical structures.

With all the inner anatomy structures segmented, the complete 3D model is obtained, for later fusion with the 3D thermal outer shell. The result showing the 3D reconstruction and visualization of all the structures combined, including the inner structures and the external geometry of the neck, is shown in Figure 17.

**Figure 18:**
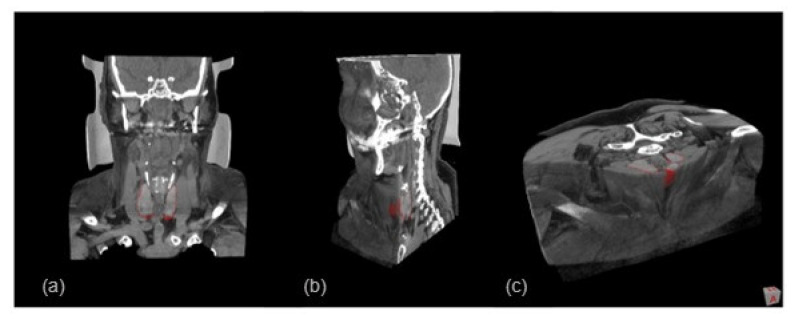
Anatomy images (DICOM) showing the corresponding 3D visualization in MeVisLab. Special attention is given to the detail of the segmented thyroid region (represented in red), within the three views: (**a**) coronal, (**b**) sagittal and (**c**) axial.

As an illustration of the result of the anatomical region by viewing only the anatomical slices (in DICOM format), Figure 18 shows the three cross-sections obtained through the CT images (coronal, sagittal, and axial), which are visualized in MeVisLab.

### 4.2. Generation of 3D Thermal Model: 3D Registration and Alignment

Since the 3D model of the outer geometry is obtained from an anatomy (CT) image, the obtained 3D model is considered a gold standard geometry. Then, after the infrared images are coupled and aligned with the 3D shell, the results from the 3D thermal shell (including the thermal texture) are obtained and then evaluated. After the alignment, the images are projected and represented by visualizing the inherent texture and thermal variations, generating the 3D thermal shell, as shown in Figure 11 (column 4).

### 4.3. Generation of 3D THERMO-SCAN Model: Complete 3D Visualization

**Figure 19:**
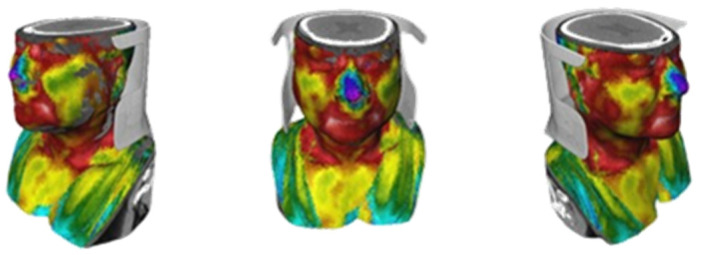
The results were visualized after the fusion between the 3D thermal shell (external) with the DICOM data (internal).

The final visualization of the complete 3D model obtained using the MeVisLab^®^ software demonstrates the fusion and registration between the 3D thermal shell and the DICOM data (i.e., the CT anatomical images), presented in Figure 19.

Therefore, this 3D THERMO-SCAN methodology, originally developed by our research group, enables a 3D visualization using a specific interface for this purpose, the MeVisLab^®^. This interface allows 3D visualization, including several imaging modalities: infrared texture outer shell, 3D inner geometries, and anatomical images (DICOM files)—all of which are registered and combined into a unique visualization.

**Figure 20:**
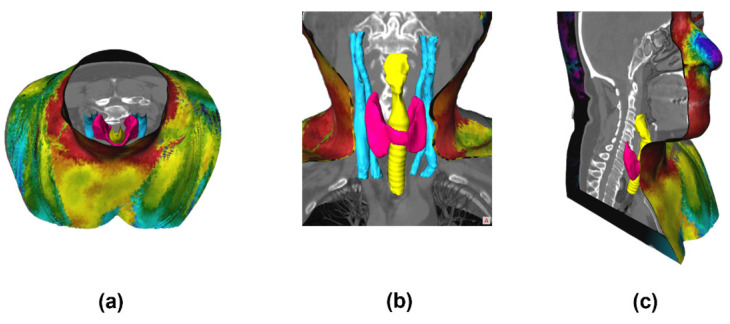
3D visualization of the complete model, showing the structures in different sections/slices: (**a**) axial, (**b**) coronal, and (**c**) sagittal. A video overview illustrating these results can be found in the Supplementary Material (Video S1).

On the inside of the complete 3D model, a set of segmented structures are presented where it is possible to observe the following structures: thyroid (pink), trachea (yellow), and veins and arteries (blue) (as seen in Figure 20). Also, a video overview illustrating these results can be found in the Supplementary Information (Video S1). These inner colors were chosen to highlight and emphasize the anatomical region under analysis. These views are obtained through the *View3D* module, to control the cuts through the *SoClipPlane* module in the MeVisLab^®^ software.

The main advantage of MevisLab^®^ is that it offers a complete toolset, including a variety of digital image processing methods, as well as providing libraries capable of processing DICOM format files (i.e., anatomy images). By means of connectable modules, the algorithms and applications are developed through networks of functional units, in which a module represents a method to be applied to the input data (image). Figure 13 represents the network of modules employed in this research. Still, the networks can be encapsulated in macro modules to be reused in other algorithms or take part in customized applications [46]. 

Additionally, by using the software MeVisLab^®^, the generated 3D THERMO-SCAN model is filled (incorporated) internally with the segmented structures, enabling visualization of the reconstructed structures from different types of files. In this case, it was possible to add more than one 3D object, which will be visualized together in the same coordinate system (such as the 3D thermal outer shell/geometry, the DICOM imaging slices, and the segmented structures, e.g., thyroid, trachea, veins, and arteries).

This visualization interface (MeVisLab) has the differential for the joint visualization of the external layer (that is, the thermal shell with the superimposed texture), with the anatomical structures (internally) already previously segmented (through CT images/slices), and with the inclusion of the DICOM anatomy imaging slices itself.

**Figure 21:**
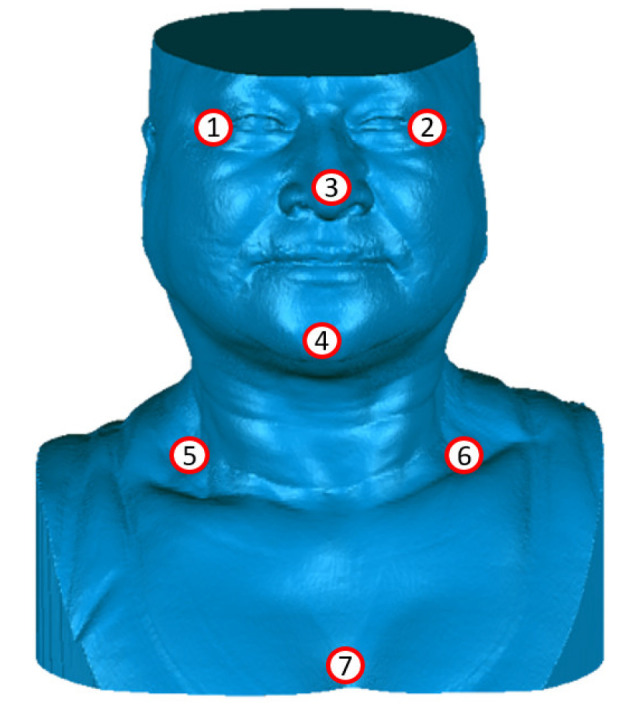
Positioning markers representing the seven points selected for quantification.

Additionally, with the case study presented here, in each 3D model (both 3D thermal and 3D geometry), seven positions spread all over the model were independently selected, as indicated in Figure 21. Then, the displacement error of these spatial positions (XYZ) was calculated based on their Euclidean distance (as presented in Table 1). It obtained an average error of 1.77 mm, which is considered reasonable, especially for the size of the subject being imaged (covering the head, neck, shoulders, and torso). According to Vasavada et al. [47], the average neck width is about 106 mm and the head width about 148 mm (for female adults averaging 1690 mm height and 66 kg body weight).

Such measurements were inspired by the previous research by Krefer et al. [18], in which an average error of 4.58 mm was obtained for the four subjects imaged. On the other hand, when using a test object, 1.41 mm was obtained. Therefore, this initial quantification represents the efficacy of the proposed methodology, which could be expanded by other medical applications related to infection, inflammation, and different pathologies (which provide more temperature variations), achieving accurate diagnoses and follow-ups.

## 5. Discussion

This research describes the generation of complete 3D THERMO-SCAN models (i.e., a customized methodology), including the imaging modalities originally presented: functional (infrared) and anatomy (CT) images. The results were available by using both imaging modalities and their corresponding computational processing, together with the visualization tools (performed using SfM methodology, affine alignment, MIMICS, MeshLab and MeVisLab software). The process consists of several steps, covering the imaging acquisition, segmentation of the CT anatomical images, and processing the infrared functional images to combine and unify them into a single and unique 3D model.

The segmentation of anatomical images (DICOM) is one of the first stages of image processing. There are automatic segmentation methods, but the most modern ones allow user interaction with the software. It is possible to define what should be extracted from each DICOM slice [48].

According to Grady [48], an interactive segmentation algorithm must have four qualities: fast processing, fast editing, the ability to segment the image by itself with enough user interactions, and finally, it must offer an intuitive segmentation process. The segmentation must be performed in software where these manipulations are possible, as the 3D structures will be highlighted when the 3D thermal geometry shell and the anatomical images are joined together. For this reason, the MIMICS software was chosen.

After the anatomic segmentation, the SfM methodology employs infrared images for generating a 3D point cloud, which is based on the calculation of the intrinsic 3D positioning coordinates (X, Y, Z). Then, this mapping/modeling of the body (volunteer) not only generates the point cloud of the object, but also obtains the positioning of the cameras in the space. The generation of the 3D thermal outer shell needs to be performed using an alignment due to the reason that we have two different imaging modalities. Such images are obtained within two different imaging acquisitions: CT images (anatomy imaging modality) and infrared images (functional imaging modality), which are acquired by different equipment at different moments. That is why it is crucial to perform such alignment between such different imaging modalities.

The initial 3D geometry obtained is a hollow model, showing only the outer shell (Figure 12a), which does not contain internal organs and structures, nor present thermal differences and color palette variations. However, within the inclusion of the DICOM images (Figure 12), the inner anatomic structures information is obtained. For these reasons, the alignment is also necessary, i.e., to keep everything together in a common coordinate system (which is the DICOM imaging system). On the other hand, infrared thermal images are located in a different coordinate system. So, after the infrared imaging is projected onto the 3D shell, the 3D thermal model is finally generated (Figure 12b), which is already located in the DICOM system to unify it all. 

In this research, we proposed a similar methodology to Krefer et al. [18], which computes 3D thermal models by employing pose estimation via Structure from Motion (SfM). However, in their work, only the generation of the 3D thermal outer shell was shown. On the other hand, Schollemann et al. [31] pointed to an additional approach to generate 3D anatomical thermal models based on multi-modality imaging, combining (outer and inner) from both infrared images and CT data, but in [31], no inner segmented anatomical structures were presented. Therefore, this is the contribution of this research: a 3D thermal anatomy model with the visualization of segmented inner organs inside it, together with the anatomy single imaging slices.

In this paper, the final visualization and fusion of different medical imaging modalities is performed using MeVisLab. The use of such software is justified based on several recent studies in the medical field, such as De buck et al. [49], Hernandez et al. [50], Liu et al. [51], Chen et al. [52], Regnstrand et al. [53], and Egger et al. [54]. Additionally, the use of MeVisLab enables further processing tools for developers as well, including customized programing in C++ and Python. 

Therefore, this paper brings the inclusion of multi-modality imaging systems, such as infrared thermography, CT anatomical DICOM images, and the correspondent segmented anatomy regions of interest. All these data, including the complete 3D thermal models, are combined and visualized into a common coordinate system to be presented altogether.

## 6. Conclusions

This paper presents the generation of complete 3D thermal anatomy models based on the methodology known as 3D THERMO-SCAN, which includes different imaging modalities. The images being used are infrared thermal and anatomical CT images. For the processing, different computational tools were employed: MATLAB, MIMICS, Visual SfM, MeshLab and MeVisLab software—enabling the 3D reconstruction and visualization of both imaging modalities into a unique and unified 3D model, combining functional and anatomy images altogether. 

This research showed results on the thyroid and neck region, demonstrating that combined functional and anatomical images have great relevance for medicine related to diagnosing and monitoring pathologies over time. It can also be considered that this methodology has great potential for further expansion, as it could be applied to other organs (parts) of the human body.

Within the proposed case study, an evaluation of the 3D thermal geometry was performed, achieving 1.77 mm precision (when compared with the CT geometry itself). Therefore, the methodology was considered reasonable, regarding the close-range imaging acquisition of a whole human head/bust/neck and the combination of different imaging modalities (i.e., infrared thermal images and CT medical data), acquired at different moments. This solution involves a decoupled acquisition mainly due to the limitation of including external interferences in the clinical anatomy CT or MRI imaging acquisitions—as it is neither allowed nor feasible to include infrared acquisitions during a CT/MRI scanning process. 

Then, this represents a valuable solution, especially when we are unable to simultaneously collect data. These solutions may support the evolution of medicine, allowing the inclusion of additional exams (using different technologies and modalities), such as different upcoming imaging modalities. For that reason, with the combination and fusion of supplementary imaging methods, there is always the need for further processing, in order to include applications in different medical fields, allowing us to achieve fast and accurate diagnoses and follow-ups.

## Figures and Tables

**Table 1:** Displacement error for the positioning markers between the 3D geometry and the 3D thermal model.

Volunteer (Case Study)	Average Error (mm)	St. Deviation (mm)	Minimum Error (mm)	Maximum Error (mm)
	1.77	1.06	0.28	3.39

## Data Availability

Not applicable.

## Supplementary Materials

The following supporting information can be downloaded at: https://www.mdpi.com/article/10.3390/s23031610/s1. Video S1: Overview of the results.
